# Central sensitization syndromes as a neuropsychiatric risk state for dementia: Clinical and epidemiologic perspectives

**DOI:** 10.1017/S1092852926101114

**Published:** 2026-07-29

**Authors:** Kevin V. Hackshaw

**Affiliations:** Division of Rheumatology, Department of Medicine, Dell Medical School at The University of Texas at Austin, USA

**Keywords:** Dementia risk, central sensitization, chronic pain, neuropsychiatry, depression, sleep disturbance, midlife risk, cognitive decline

## Abstract

Dementia prevention increasingly targets modifiable midlife risk states contributing to neurodegenerative vulnerability. While vascular and metabolic factors are established, a substantial proportion of risk remains unexplained. Central sensitization syndromes (CSS)—chronic conditions characterized by neural amplification—are highly prevalent, co-occur with depression and sleep disturbance, and involve neuroimmune and network-level mechanisms implicated in early Alzheimer’s disease. To synthesize epidemiologic evidence linking CSS with incident dementia and evaluate competing causal explanations, including shared vulnerability, reverse causation, and medication-related confounding. A structured narrative review was conducted of longitudinal cohort and meta-analytic studies assessing incident dementia across CSS-related conditions. Multiple CSS—including migraine, fibromyalgia, irritable bowel syndrome, restless legs syndrome, and PTSD disorder—are associated with modest-to-moderate increases in dementia risk. Multisite chronic pain demonstrates dose–response relationships with hippocampal atrophy and incident dementia. However, many conditions lack dedicated longitudinal studies. Interpretation is limited by residual confounding, reverse causation, healthcare utilization bias, and medication effects. CSS are associated with increased dementia risk but causality remains unproven. Central sensitization may represent a clinically identifiable vulnerability state integrating pain, affective burden, and sleep disruption. This highlights a potentially high-risk population that may not be fully captured by traditional dementia risk models. Biomarker-integrated longitudinal studies and advanced causal modeling are needed to clarify mechanisms.

## Introduction

Alzheimer’s disease and related dementias develop over decades and are increasingly understood as the downstream manifestation of cumulative biological vulnerability.^1^ ^–^^8^ Contemporary prevention frameworks emphasize the identification of modifiable midlife risk states that influence long-term neurodegenerative trajectories. While vascular and metabolic factors are well established, they do not fully account for the heterogeneity of cognitive aging observed across populations.^9^ ^–^^17^ This gap has prompted increasing interest in systemic conditions that may contribute to cumulative central nervous system stress and reduced resilience over time. From a neuropsychiatric perspective, central sensitization syndromes (CSS) may represent a convergent clinical phenotype in which pain, mood, sleep, and cognitive symptoms co-occur, reflecting shared network-level vulnerability relevant to neurodegenerative risk.

Interpretation of dementia risk in CSS is complicated by extensive pharmacologic exposure. Individuals with these conditions frequently receive long-term pharmacotherapy targeting pain amplification, sleep disturbance, mood symptoms, and autonomic dysregulation.^15^ ^–^^17^ Many of these medications—including anticholinergic agents, benzodiazepines, sedative-hypnotics, gabapentinoids, and certain antidepressants—have potential cognitive effects or have been associated with dementia risk in observational studies of older adults. Consequently, associations between CSS and dementia may reflect not only underlying disease biology but also medication exposure patterns, polypharmacy, and confounding by indication. Clarifying these relationships is therefore essential for interpreting epidemiologic findings and for guiding safer pharmacologic management of chronic pain in aging populations.

CSS are chronic conditions characterized by amplified central nervous system processing of sensory and interoceptive input.^18^ ^–^^22^ Though historically framed within pain medicine, CSS extend beyond nociceptive amplification and encompass fatigue, cognitive complaints, sleep disturbance, autonomic dysregulation, and affective symptoms.^23^ ^–^^27^ Core CSS include fibromyalgia, migraine, irritable bowel syndrome, temporomandibular disorders, interstitial cystitis/bladder pain syndrome, vulvodynia, and chronic multisite pain. Additional conditions such as post-traumatic stress disorder (PTSD), restless legs syndrome, endometriosis, dysmenorrhea, PCOS, and multiple chemical sensitivity may exhibit sensitization-related mechanisms but occupy a less established position within the CSS construct.^28^ ^–^^33^

CSS share convergent biological themes: neuroimmune priming, glial activation, altered salience and default mode network connectivity, hypothalamic–pituitary–adrenal (HPA) axis dysregulation, metabolic disturbance, and chronic sleep disruption.^34^ ^–^^36^ These mechanisms overlap substantially with emerging models of early AD vulnerability.^37^ ^–^^39^

Epidemiologic studies increasingly report elevated incident dementia risk among individuals with several CSS.^40^ ^–^^47^ However, interpretation is complicated by overlapping comorbidities, medication exposure, and potential reverse causation.^48^ ^–^^51^ Recent work in fibromyalgia has further emphasized the interconnected nature of central sensitization, depressive burden, functional impairment, and neurobehavioral vulnerability. Data-driven phenotyping studies demonstrate stable symptom clusters characterized by varying levels of pain, depression, and central sensitization, suggesting that sensitization-related disorders may reflect broader neurobehavioral states rather than isolated pain syndromes. This conceptual framework supports investigation of central sensitization as a clinically identifiable vulnerability phenotype relevant to long-term brain health.^52^ ^–^^54^ This review synthesizes epidemiologic evidence linking CSS with incident dementia and evaluates competing causal explanations using a structured framework. Clinical and research implications are outlined relevant to dementia risk stratification and prevention.

## Methods

A structured narrative review was conducted informed by epidemiologic principles of causal inference and bias assessment of the epidemiologic and mechanistic literature. Searches of PubMed/MEDLINE and Scopus were performed through January 2026 using condition-specific terms combined with “dementia,” “Alzheimer’s disease,” “incident,” “longitudinal,” and “cohort. Searches incorporated combinations of controlled vocabulary terms (Medical Subject Headings [MeSH] where applicable) and free-text keywords. Representative PubMed search syntax included combinations of (“fibromyalgia” OR “central sensitization” OR “migraine” OR “irritable bowel syndrome” OR “restless legs syndrome” OR “post-traumatic stress disorder”) AND (“dementia” OR “Alzheimer disease” OR “cognitive decline”) AND (“cohort” OR “longitudinal” OR “incident”). Equivalent database-specific syntax was used for Scopus. English-language studies were included. Reference lists of eligible articles and relevant reviews were manually screened to identify additional studies. Included are prospective cohorts, retrospective population-based cohorts, nested case–control analyses, and meta-analyses reporting incident dementia. Cross-sectional cognitive studies were excluded from the incidence synthesis. Studies evaluating cognitive decline, mild cognitive impairment, neuroimaging markers, hippocampal atrophy, or dementia biomarkers without incident dementia outcomes were not included in evidence grading but were reviewed separately as mechanistic or supportive evidence. All-cause dementia and Alzheimer disease-specific outcomes were recorded separately whenever reported.

Adjusted hazard ratios (HR), relative risks (RR), or odds ratios (OR) were extracted where available. Alzheimer’s disease–specific endpoints were prioritized when reported.

Confounding by indication refers to bias arising when medications are prescribed because of disease severity or characteristics that themselves influence dementia risk. Protopathic bias occurs when treatment is initiated for symptoms that are actually early manifestations of an undiagnosed disease.

Strength of Evidence Classification:

1. Moderate-to-high (replicated cohorts or meta-analyses)
2. Moderate (single large cohort)
3. Evidence gap (absence of dedicated longitudinal data)
  Literature screening and data extraction were conducted by the author. Eligible studies were reviewed in full text. Data extracted included study design, population, follow-up duration, dementia outcome definition, adjustment variables, and effect estimates. Evidence classifications assigned to each condition are summarized in Table 1. A structured synthesis of epidemiologic studies, including effect estimates, adjustment variables, and evidence ratings, is provided in Table 2.

**Table 1.** Evidence Classification of Dementia Risk Across Central Sensitization Syndromes Table 1. long description.

Condition	Evidence Category
Multisite Chronic Pain	Moderate-High
Migraine	Moderate-High
PTSD	Moderate-High
Fibromyalgia	Moderate
IBS	Moderate
RLS	Moderate
ME/CFS	Evidence Gap
TMD	Evidence Gap
CRPS	Evidence Gap
IC/BPS	Evidence Gap
Vulvodynia	Evidence Gap
Endometriosis	Evidence Gap
PCOS	Evidence Gap
Dysmenorrhea	Evidence Gap
MCS	Evidence Gap

Evidence classifications were assigned based on the quantity, consistency, and methodological quality of available longitudinal studies. Moderate-to-high evidence indicates support from replicated cohort studies and/or meta-analyses. Moderate evidence indicates support from one or more large observational cohorts with consistent findings but limited replication. Evidence gap indicates absence of dedicated longitudinal studies evaluating incident dementia outcomes.

**Table 2.** Epidemiologic Evidence Linking Central Sensitization Syndromes to Incident Dementia Table 2. long description.

Condition	Key Studies	Design	N	Outcome	Effect Estimate	Depression Adjusted	Sleep Adjusted	Medication Adjusted	Lag Period Used	Evidence Rating
Fibromyalgia	Chang 2016; Lin 2017; Tzeng 2018	National cohort	>40 000	All-cause dementia	HR 1.45–2.77	Partial	No	Limited	Rare	Moderate
Migraine	Zhang 2021 meta-analysis	Meta-analysis	>1 million	All-cause dementia	RR ~1.30	Variable	Rarely	Rarely	Variable	Moderate-High
IBS	Lee 2016; Tsai 2017; Fang 2022	Cohort/meta-analysis	>100 000	Dementia/AD	HR 1.20–1.50	Partial	No	No	Rare	Moderate
RLS	Yoo 2020; Kim 2020	National cohort	>50 000	All-cause dementia	HR 1.20–1.80	Variable	Limited	No	Rare	Moderate
PTSD	Yaffe 2010; Zhang 2018	Cohort/meta-analysis	>200 000	Dementia	RR 1.50–2.00	Usually	Limited	Limited	Variable	Moderate-High
Multisite Chronic Pain	Veronese 2017; Wang 2019; Shen 2021; Zhao 2023	Prospective cohort	>400 000	Dementia + imaging	HR 1.30–2.00	Yes	Variable	Rarely	Some cohorts	Moderate-High
ME/CFS	None	–	–	–	–	–	–	–	–	Evidence Gap
TMD	None	–	–	–	–	–	–	–	–	Evidence Gap
CRPS	None	–	–	–	–	–	–	–	–	Evidence Gap
IC/BPS	None	–	–	–	–	–	–	–	–	Evidence Gap
Vulvodynia	None	–	–	–	–	–	–	–	–	Evidence Gap
Endometriosis	None	–	–	–	–	–	–	–	–	Evidence Gap
PCOS	None	–	–	–	–	–	–	–	–	Evidence Gap
Dysmenorrhea	None	–	–	–	–	–	–	–	–	Evidence Gap
MCS	None	–	–	–	–	–	–	–	–	Evidence Gap

Summary of representative longitudinal cohort studies and meta-analyses evaluating incident dementia risk across central sensitization syndromes and related conditions. Effect estimates represent adjusted hazard ratios (HR), relative risks (RR), or odds ratios (OR) reported in the literature. Adjustment columns indicate whether studies accounted for depression, sleep-related factors, or medication exposure. Lag period refers to exclusion periods used to reduce reverse-causation bias. Evidence ratings reflect overall strength of the available epidemiologic evidence.

### Causal inference framework

**Figure 1.**
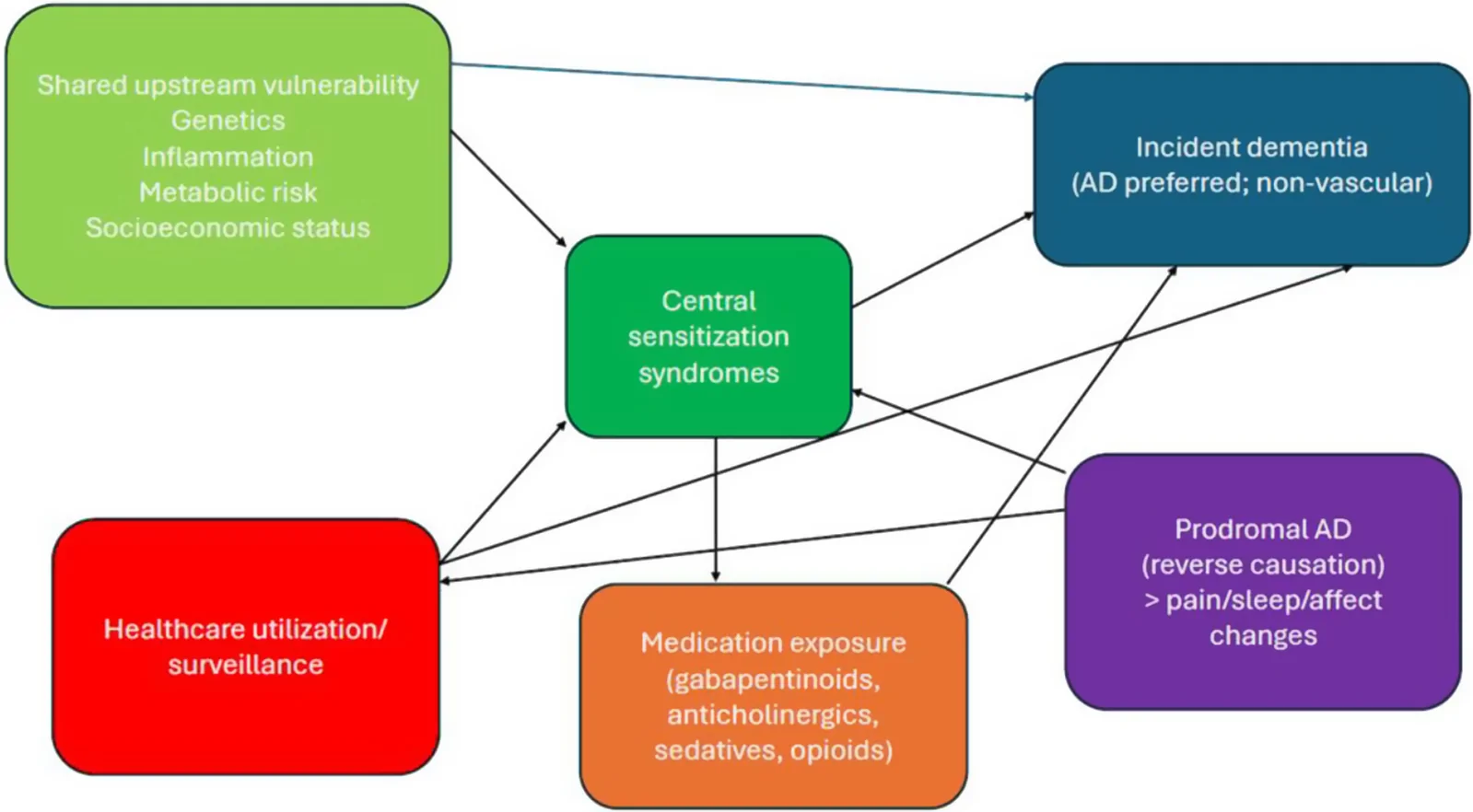
Directed acyclic graph. Directed acyclic graph (DAG) illustrating hypothesized relationships between central sensitization syndromes (CSS) and incident dementia. Pathways include direct neurobiological effects, mediation through depression, sleep disturbance, and chronic pain, and confounding by shared vulnerability factors. Reverse causation, medication-related effects, and healthcare utilization bias are also depicted, highlighting competing causal explanations. Figure 1. long description.

Observed associations between CSS and incident dementia should be interpreted within explicit causal frameworks rather than as inherently directional effects. One conceptual model proposes that CSS may be associated with sustained neuroimmune activation, which could contribute to network-level dysfunction and pathways relevant to AD vulnerability. Chronic neuroimmune activation may alter synaptic plasticity, promote maladaptive glial signaling, disrupt salience-default mode network interactions, impair hippocampal function, and reduce neural resilience. These processes overlap with biological pathways implicated in early neurodegenerative vulnerability, although direct longitudinal mediation remains unproven. In this model, persistent peripheral and central sensitization amplify glial activation and inflammatory signaling, lowering the threshold for synaptic dysfunction and accelerating vulnerability within memory-relevant neural circuits. This pathway is biologically plausible and aligns with converging evidence linking chronic inflammation, synaptic dysregulation, and neurodegenerative progression^22^ ^,^^34^ ^,^^37^ (Figure 1).

However, several alternative causal structures must also be considered. One possibility is shared upstream vulnerability: systemic inflammation, cardiometabolic risk, genetic susceptibility, or stress-related neuroendocrine dysregulation could independently increase the likelihood of both CSS and dementia, creating a non-causal association driven by common antecedents. A second explanation is reverse causation, whereby prodromal AD—long before overt cognitive symptoms emerge—alters pain processing networks and increases pain sensitivity, thereby manifesting clinically as a sensitization phenotype. Third, differential healthcare utilization may introduce surveillance bias in observed relationships; individuals with chronic pain conditions often have more frequent medical contact, increasing the probability of earlier cognitive assessment and diagnosis. Finally, medication-mediated pathways must be addressed, as commonly prescribed agents for chronic pain, mood disturbance, or sleep disruption may themselves influence cognitive trajectories either positively or negatively.^12^ ^,^^14^ Without study designs capable of distinguishing among these competing pathways—through temporally anchored exposures, biomarker integration, and advanced causal modeling—firm etiologic inferences regarding CSS as a driver of dementia risk cannot be justified.^50^ These considerations highlight that associations between chronic sensitization states and dementia risk should be interpreted within an explicit life-course framework in which exposures, mediators, and prodromal disease processes evolve dynamically over time. Framing these relationships within psychiatric epidemiology emphasizes that chronic pain, mood disorders, and sleep dysregulation should be modeled jointly rather than as isolated exposures in dementia risk research.

### Condition-specific evidence

The overall strength of evidence across CSS is summarized in Table 1, while key epidemiologic studies and methodological characteristics are summarized in Table 2.

### Fibromyalgia

Population-based cohort studies report elevated dementia risk among individuals with fibromyalgia, with adjusted HR generally ranging from approximately 1.4 to 2.0 depending upon cohort characteristics and outcome definitions.^23^ ^,^^24^ ^,^^27^ ^,^^40^ ^,^^47^ ^,^^55^ ^–^^57^ Taiwanese nationwide studies reported adjusted HRs ranging from 1.45 to 2.77 for incident dementia during follow-up periods of approximately 5–10 years. Some analyses demonstrate AD-specific associations after multivariable adjustment.^40^ ^,^^47^ ^,^^55^ However, FM populations exhibit high prevalence of depression, sleep disturbance, metabolic risk, and psychotropic medication exposure. Few studies incorporate extended lag periods or biomarker-confirmed AD endpoints.^1^ ^,^^5^ ^,^^40^ ^,^^55^ Consequently, whether FM represents a causal contributor, shared vulnerability marker, or early manifestation of prodromal AD remains unresolved. Evidence rating: Moderate.

### Migraine

Meta-analyses demonstrate modest but consistent increased dementia risk among migraineurs.^58^ ^–^^61^ Vascular and inflammatory mechanisms may mediate risk, but antidepressant exposure and healthcare utilization confound interpretation.^62^ ^–^^65^ Time-varying models and AD biomarker integration are needed. Meta-analytic estimates suggest pooled RR in the range of 1.25–1.40 for incident dementia. Evidence rating: Moderate-to-high.

### Irritable bowel syndrome

IBS cohorts demonstrate elevated dementia incidence, including AD-specific signals.^66^ ^–^^68^ Gut–brain axis dysregulation provides mechanistic plausibility; however, psychiatric comorbidity and metabolic clustering remain substantial confounders.^69^ ^,^^70^ Cohort studies generally report adjusted HRs between 1.2 and 1.5. Evidence rating: Moderate.

### Restless legs syndrome

RLS is associated with increased dementia risk in retrospective cohorts.^71^ ^–^^73^ Chronic sleep fragmentation offers a biologically plausible pathway, yet dopaminergic medication exposure complicates interpretation.^74^ ^,^^75^ Available retrospective cohorts report adjusted HRs ranging from approximately 1.2 to 1.8. Evidence rating: Moderate.

### Post-traumatic stress disorder

Meta-analytic evidence supports elevated dementia risk in PTSD.^76^ ^–^^79^ Chronic glucocorticoid exposure and hippocampal vulnerability are plausible mediators, but reverse causation cannot be excluded.^80^ ^–^^82^ Meta-analyses generally report pooled risk estimates between 1.5 and 2.0. Evidence rating: Moderate.

### Chronic multisite pain

Prospective cohorts demonstrate dose–response relationships between number of pain sites and dementia risk, strengthening causal plausibility under Hill criteria.^83^ ^–^^85^ Neuroimaging analyses further demonstrate associations with hippocampal atrophy and accelerated cognitive decline. Nevertheless, shared inflammatory or metabolic vulnerability may underlie both. The UK Biobank analysis by Zhao et al. demonstrated a dose–response relationship between increasing pain-site burden and incident dementia risk. Individuals reporting four or more pain sites exhibited significantly elevated dementia incidence compared with participants without chronic pain. Associations persisted after adjustment for demographic characteristics, vascular risk factors, depression, and socioeconomic variables. Neuroimaging analyses further demonstrated smaller hippocampal volumes and accelerated cognitive decline among individuals with greater multisite pain burden. Evidence rating: Moderate-to-high.

### ME/CFS, CRPS, TMD, vulvodynia, MPS, IC/BPS, dysmenorrhea, PCOS, endometriosis, MCS

For several CSS—including temporomandibular disorders, complex regional pain syndrome, myalgic encephalomyelitis/chronic fatigue syndrome, vulvodynia, myofascial pain syndrome, interstitial cystitis/painful bladder syndrome, primary dysmenorrhea, polycystic ovarian syndrome, endometriosis, and multiple chemical sensitivity—dedicated longitudinal dementia-incidence studies are sparse or absent. Nevertheless, these conditions share biological themes with better-studied CSS, including neuroimmune activation, autonomic imbalance, sleep disturbance, metabolic dysfunction, and high psychiatric comorbidity. Although mechanistic overlap provides biologic plausibility, there is currently insufficient longitudinal evidence to infer elevated dementia risk for these conditions. Accordingly, these disorders should be viewed as evidence gaps rather than established dementia-risk phenotypes.^31^ ^,^^86^ ^–^^94^

## Limitations of the current evidence

### Depression as a major confounding and mediating factor

Depression represents a particularly important potential confounder in studies examining associations between CSS and dementia risk.^16^ ^,^^95^ ^–^^97^ Major depressive disorder occurs at substantially higher prevalence among individuals with many CSS, including fibromyalgia, migraine, irritable bowel syndrome, and chronic multisite pain. Shared biological mechanisms—including chronic stress exposure, HPA axis dysregulation, neuroimmune activation, altered monoaminergic signaling, and sleep disturbance—likely contribute to this comorbidity.^21^ ^,^^27^ ^,^^98^ ^–^^101^ As a result, depression may represent both a shared vulnerability state and a downstream consequence of chronic sensitization.

Independently of chronic pain conditions, depression itself has been consistently associated with increased risk of incident dementia in large population-based cohorts and meta-analyses.^102^ ^–^^106^ Individuals with a history of major depressive episodes demonstrate significantly elevated risk of subsequent cognitive decline and Alzheimer’s disease compared with non-depressed controls. Several mechanisms have been proposed to explain this relationship, including chronic glucocorticoid exposure leading to hippocampal atrophy, sustained neuroinflammatory signaling, reduced neurotrophic support, vascular injury, and behavioral mediators such as reduced physical activity and social engagement.^107^ ^–^^111^

Importantly, depression may operate in multiple causal roles within CSS–dementia associations. First, depression may act as a confounder, increasing the likelihood of both sensitization-related disorders and later dementia. Second, depression may function as a mediator, arising as a consequence of chronic pain and contributing to downstream neurodegenerative vulnerability. Third, depressive symptoms may represent an early manifestation of prodromal neurodegeneration, further complicating causal interpretation.^112^ ^–^^115^ Without longitudinal study designs that incorporate time-varying measures of mood symptoms and adequate lag periods, disentangling these pathways remains difficult. Future studies examining sensitization-related dementia risk should therefore incorporate detailed assessment of depressive symptom trajectories and consider analytic approaches capable of distinguishing confounding from mediation.

### Sleep disturbance and sleep-disordered breathing as shared risk pathways

Sleep disturbance represents another important potential confounder in studies examining associations between CSS and dementia risk. Disrupted sleep architecture is highly prevalent across many CSS, including fibromyalgia, migraine, irritable bowel syndrome, restless legs syndrome, and PTSD.^21^ ^,^^36^ ^,^^39^ ^,^^116^ ^,^^117^ Chronic insomnia, fragmented sleep, circadian disruption, and sleep-disordered breathing may therefore represent shared biological vulnerabilities rather than downstream consequences of sensitization alone.

Accumulating evidence suggests that impaired sleep contributes directly to neurodegenerative processes relevant to Alzheimer’s disease.^118^ ^–^^121^ Experimental and longitudinal studies demonstrate that reduced slow-wave sleep and sleep fragmentation impair glymphatic clearance of amyloid-β and tau from the interstitial space, potentially accelerating accumulation of pathogenic proteins within the brain.^122^ ^–^^124^ Obstructive sleep apnea further introduces intermittent hypoxia, oxidative stress, endothelial dysfunction, and systemic inflammation—mechanisms that may amplify neurodegenerative vulnerability.^125^ ^–^^128^

Notably, sleep disturbance may operate in several causal roles within CSS–dementia associations. First, poor sleep may function as a confounder, increasing the likelihood of both chronic sensitization states and later dementia.^129^ ^,^^130^ Second, sleep disruption may serve as a mediator, arising as a consequence of chronic pain or autonomic dysregulation and subsequently promoting neurodegenerative processes.^131^ ^,^^132^ Third, alterations in sleep architecture may represent an early manifestation of prodromal Alzheimer’s disease, further complicating causal interpretation.^133^ ^,^^134^ Without longitudinal studies incorporating objective sleep measurements and extended lag periods, disentangling these pathways remains challenging.

Future studies examining sensitization-related dementia risk should therefore incorporate detailed characterization of sleep architecture, sleep-disordered breathing, and circadian disruption. Integration of polysomnography, actigraphy, and sleep biomarkers may help clarify whether disturbed sleep represents a causal pathway linking sensitization states to neurodegenerative risk or instead reflects shared upstream vulnerability.

Most studies rely on administrative data with ICD-coded exposure and outcome definitions. Exposure and outcome misclassification are likely. Few datasets incorporate biomarker-confirmed AD endpoints.

### Medication exposure and pharmacoepidemiologic confounding

Medication exposure introduces a fundamental challenge in interpreting associations between CSS and dementia risk, as it operates within complex, time-varying causal structures. Pharmacologic treatments for chronic pain, sleep disturbance, and affective symptoms are not randomly assigned but are instead closely linked to disease severity, comorbidity burden, and healthcare utilization. As a result, observed associations between medication use and dementia may reflect confounding by indication, reverse causation, or cumulative disease burden rather than direct pharmacologic effects. From a causal inference perspective, medication exposure should therefore be conceptualized as a dynamic mediator-confounder within longitudinal pathways linking sensitization states to neurodegenerative outcomes, requiring analytic approaches that explicitly account for time-dependent confounding.^135^ ^–^^138^ Active-comparator designs compare treatments prescribed for similar indications, helping reduce confounding by indication.

Gabapentinoids, including gabapentin and pregabalin, are widely prescribed for neuropathic and centralized pain states. Although generally considered cognitively neutral in short-term clinical trials, emerging pharmacoepidemiologic studies have raised concerns regarding possible associations between chronic gabapentinoid exposure and increased dementia incidence in some populations.^139^ ^–^^141^ Interpretation of these findings remains challenging because gabapentinoids are preferentially prescribed to individuals with severe chronic pain, multimorbidity, and polypharmacy—factors that themselves may confer elevated dementia risk. Distinguishing medication-related effects from underlying disease severity therefore requires analytic approaches capable of addressing confounding by indication.

Anticholinergic medications represent another important potential confounder in this literature. Drugs with anticholinergic properties—including tricyclic antidepressants, certain bladder antispasmodics, first-generation antihistamines, and several psychotropic agents—are commonly used in patients with chronic pain, sleep disturbance, and autonomic symptoms. A substantial body of epidemiologic research has demonstrated associations between cumulative anticholinergic exposure and increased risk of cognitive decline and dementia.^142^ ^–^^145^ Because many CSS patients receive medications with moderate or strong anticholinergic burden, medication exposure may contribute to observed associations between sensitization syndromes and dementia risk independent of the underlying disorder.

Additional medication classes frequently prescribed in CSS populations may also influence cognitive trajectories. Benzodiazepines and sedative-hypnotics are commonly used for insomnia and anxiety but have been linked to cognitive impairment and increased dementia risk in some observational studies.^146^ ^–^^148^ Opioids, although less central to contemporary management of centralized pain, may contribute to cognitive dysfunction through sedation, sleep disruption, and altered neuroinflammatory signaling.^149^ ^–^^151^ Antidepressants—particularly those with strong anticholinergic properties—may similarly influence cognitive outcomes, although evidence remains mixed and may reflect underlying depression severity rather than medication-specific effects.

Polypharmacy further complicates interpretation of epidemiologic findings. Individuals with chronic multisymptom conditions frequently receive multiple medications affecting central nervous system signaling, autonomic function, and sleep regulation. Interactions among these agents may produce cumulative cognitive effects that are difficult to disentangle in administrative datasets lacking detailed dosage and duration information.

Future studies examining sensitization-related dementia risk should incorporate pharmacoepidemiologic strategies capable of addressing these complexities. New-user cohort designs, active comparator analyses, cumulative exposure modeling, and time-varying medication covariates can help mitigate confounding by indication and immortal time bias. Without such methodological approaches, medication exposure will remain an important source of residual confounding in studies linking CSS with dementia risk.

Residual confounding remains pervasive. Sleep architecture, inflammatory burden, socioeconomic status, and physical activity are rarely measured with sufficient precision. Protopathic bias is insufficiently addressed. Short exclusion windows do not mitigate decades-long preclinical AD pathology. Repeated longitudinal measures of sensitization severity and neurodegenerative biomarkers are largely absent.

Accordingly, future studies should prioritize pharmacoepidemiologic designs capable of disentangling these effects, including new-user cohorts, active comparator strategies, and marginal structural models with time-varying exposures. Without such approaches, medication use will remain an important source of residual bias, limiting the ability to distinguish whether observed dementia risk is attributable to underlying sensitization biology, treatment effects, or their interaction over the life course.

### Future directions

From a psychiatric epidemiology perspective, these findings highlight the importance of integrating chronic pain, affective disorders, and sleep dysregulation into unified models of neurodegenerative risk, rather than treating these domains as independent contributors. This integrated perspective aligns with contemporary models of multimorbidity in mental health, where interacting biological and behavioral domains jointly influence long-term cognitive outcomes. Rather than acting as isolated risk domains, chronic pain, depressive symptoms, sleep disturbance, and medication burden may represent interacting components of a shared vulnerability network. Future studies should model these domains simultaneously rather than treating them as independent covariates. Clarifying etiologic directionality between chronic sensitization states and dementia risk will require methodologic designs that move beyond traditional observational associations. First, future cohorts should integrate blood-based Alzheimer’s disease biomarkers—such as plasma Aβ42/40 ratios, phosphorylated tau (p-tau181 or p-tau217), and neurofilament light chain—to distinguish preclinical neurodegeneration from downstream cognitive outcomes.^152^ ^–^^154^ Embedding these biomarkers within pain and sensitization cohorts will allow investigators to determine whether heightened sensitization precedes measurable neurodegenerative pathology or instead reflects early central nervous system vulnerability. Second, extended lag analyses of at least 5–10 years are essential to mitigate reverse causation, particularly given the long prodromal phase of Alzheimer’s disease and related dementias.^2^ ^,^^5^ ^,^^10^ ^,^^11^ By excluding events occurring within early follow-up windows, investigators can better assess whether sensitization phenotypes represent antecedent risk states rather than early manifestations of neurodegeneration.

Quasi-experimental epidemiologic approaches will also be critical. Target trial emulation designs can explicitly define eligibility criteria, exposure assignment, follow-up windows, and outcome ascertainment in a manner that mimics randomized trials using observational data, thereby reducing immortal time and selection biases. Marginal structural models can address time-varying confounding, especially where pain severity, medication exposure, mood disorders, and healthcare utilization evolve dynamically over time and may both influence and be influenced by dementia risk. Active-comparator pharmacoepidemiologic strategies—comparing therapeutics with similar indications but differing mechanistic pathways—can further isolate medication effects from confounding by indication. Finally, repeated longitudinal assessment of sensitization severity using validated quantitative sensory testing, symptom indices, and biomarker panels will be necessary to model trajectories rather than static exposures. Capturing within-person change over time will help determine whether escalating sensitization predicts subsequent neurodegenerative biomarker conversion or cognitive decline, thereby strengthening causal inference under a life-course framework.

### Clinical implications for prescribing in older adults

Although causality has not been established, several CSS are consistently associated with increased dementia risk in observational studies. These associations highlight the importance of careful interpretation of cognitive risk signals in patients with chronic pain who often require long-term pharmacologic treatment. Particular attention should be given to minimizing cumulative anticholinergic burden, as numerous medications commonly used in these populations—including certain antidepressants, antihistamines, and bladder antispasmodics—have been associated with increased risk of cognitive decline and dementia in older adults. Similarly, prolonged benzodiazepine and sedative-hypnotic use should be approached cautiously given observational evidence linking these agents to adverse cognitive outcomes. Gabapentinoids remain widely prescribed for centralized pain states and are often considered cognitively neutral; however, emerging epidemiologic studies suggest that long-term exposure warrants continued evaluation, particularly in older patients with multimorbidity and polypharmacy. In clinical practice, these considerations support a cautious prescribing approach that prioritizes minimizing unnecessary central nervous system–active medications and incorporating periodic cognitive monitoring in patients with chronic sensitization syndromes receiving long-term pharmacotherapy.

Taken together, the available evidence supports conceptualizing CSS not as isolated clinical entities but as a composite neuropsychiatric vulnerability state. This perspective aligns with emerging models of multimorbidity in which interacting domains—pain, mood, sleep, and cognition—jointly influence long-term brain health. Framing sensitization in this manner may facilitate integration into life-course dementia risk models and guide more comprehensive clinical assessment strategies.

## Conclusions

This review positions central sensitization as a potential midlife vulnerability construct within the life-course epidemiology of dementia. Rather than representing a single causal exposure, sensitization-related conditions likely reflect interacting biological and behavioral processes that collectively shape neurodegenerative risk.

Observed associations may arise from shared vulnerability, medication effects, healthcare utilization patterns, or early manifestations of prodromal disease. Clarifying these pathways will require biomarker-integrated longitudinal studies and rigorous causal modeling. Until such evidence is available, central sensitization should be considered a clinically relevant risk phenotype rather than a confirmed causal determinant of dementia.

## Data Availability

Not applicable.

## Long descriptions

### Table 1. Long description

The table consists of two columns: Condition and Evidence Category. The data is organized as follows:

* Multisite Chronic Pain: Moderate-High

* Migraine: Moderate-High

* P T S D: Moderate-High

* Fibromyalgia: Moderate

* I B S: Moderate

* R L S: Moderate

* M E / C F S: Evidence Gap

* T M D: Evidence Gap

* C R P S: Evidence Gap

* I C / B P S: Evidence Gap

* Vulvodynia: Evidence Gap

* Endometriosis: Evidence Gap

* P C O S: Evidence Gap

* Dysmenorrhea: Evidence Gap

* M C S: Evidence Gap

Moderate-to-high evidence indicates support from replicated cohort studies or meta-analyses. Moderate evidence indicates support from large observational cohorts with limited replication. Evidence gap indicates an absence of dedicated longitudinal studies.

Navigate back to Table 1..

### Table 2. Long description

The table contains 11 columns: Condition, Key Studies, Design, N, Outcome, Effect Estimate, Depression Adjusted, Sleep Adjusted, Medication Adjusted, Lag Period Used, and Evidence Rating.

* Fibromyalgia: Studies by Chang 2016, Lin 2017, Tzeng 2018; National cohort; N greater than 40,000; Outcome: All-cause dementia; H R 1.45 to 2.77; Depression: Partial; Sleep: No; Medication: Limited; Lag: Rare; Rating: Moderate.

* Migraine: Zhang 2021 meta-analysis; N greater than 1 million; Outcome: All-cause dementia; R R approximately 1.30; Depression: Variable; Sleep: Rarely; Medication: Rarely; Lag: Variable; Rating: Moderate-High.

* I B S: Lee 2016, Tsai 2017, Fang 2022; Cohort/meta-analysis; N greater than 100,000; Outcome: Dementia/A D; H R 1.20 to 1.50; Depression: Partial; Sleep: No; Medication: No; Lag: Rare; Rating: Moderate.

* R L S: Yoo 2020, Kim 2020; National cohort; N greater than 50,000; Outcome: All-cause dementia; H R 1.20 to 1.80; Depression: Variable; Sleep: Limited; Medication: No; Lag: Rare; Rating: Moderate.

* P T S D: Yaffe 2010, Zhang 2018; Cohort/meta-analysis; N greater than 200,000; Outcome: Dementia; R R 1.50 to 2.00; Depression: Usually; Sleep: Limited; Medication: Limited; Lag: Variable; Rating: Moderate-High.

* Multisite Chronic Pain: Veronese 2017, Wang 2019, Shen 2021, Zhao 2023; Prospective cohort; N greater than 400,000; Outcome: Dementia plus imaging; H R 1.30 to 2.00; Depression: Yes; Sleep: Variable; Medication: Rarely; Lag: Some cohorts; Rating: Moderate-High.

* The following conditions are listed as Evidence Gap with no study data: M E / C F S, T M D, C R P S, I C / B P S, Vulvodynia, Endometriosis, P C O S, Dysmenorrhea, and M C S.

Navigate back to Table 2..

### Figure 1. Long description

A flowchart consisting of six colored boxes connected by directional arrows.

* At the top left, a light green box labeled Shared upstream vulnerability includes Genetics, Inflammation, Metabolic risk, and Socioeconomic status. Arrows point from this box to Central sensitization syndromes and Incident dementia.

* In the center, a dark green box labeled Central sensitization syndromes has arrows pointing to Incident dementia, Healthcare utilization forward slash surveillance, and Medication exposure.

* At the top right, a dark blue box represents the primary outcome: Incident dementia (A D preferred; non-vascular).

* At the bottom left, a red box labeled Healthcare utilization forward slash surveillance has an arrow pointing back to Central sensitization syndromes.

* At the bottom center, an orange box labeled Medication exposure (gabapentinoids, anticholinergics, sedatives, opioids) has an arrow pointing to Incident dementia.

* At the bottom right, a purple box labeled Prodromal A D (reverse causation) > pain forward slash sleep forward slash affect changes has arrows pointing to Central sensitization syndromes, Healthcare utilization forward slash surveillance, and Incident dementia.

Navigate back to Figure 1..
